# Statistical Analysis in the German National Cohort (NAKO) – Specific Aspects and General Recommendations

**DOI:** 10.1007/s10654-022-00880-7

**Published:** 2022-06-02

**Authors:** Oliver Kuss, Heiko Becher, Andreas Wienke, Till Ittermann, Stefan Ostrzinski, Sabine Schipf, Carsten O Schmidt, Michael Leitzmann, Tobias Pischon, Lilian Krist, Stephanie Roll, Matthias Sand, Hermann Pohlabeln, Stefan Rach, Karl-Heinz Jöckel, Andreas Stang, Ulrich A Mueller, Andrea Werdecker, Ronny Westerman, Karin H Greiser, Karin B Michels

**Affiliations:** 1 Institute for Biometrics and Epidemiology, German Diabetes Center, Leibniz Institute for Diabetes Research, Heinrich-Heine-University Düsseldorf, Düsseldorf, Germany; 2 Centre for Health and Society, Medical Faculty, University Hospital Düsseldorf, Heinrich-Heine-University Düsseldorf, Düsseldorf, Germany; 3 Institute for Medical Biometry and Epidemiology, University Medical Center Hamburg-Eppendorf, Hamburg, Germany; 4 Institute of Medical Epidemiology, Biometrics, and Informatics, Martin-Luther-University Halle-Wittenberg, Halle, Germany; 5 Institute for Community Medicine, University Medicine Greifswald, Greifswald, Germany; 6 Department of Epidemiology and Preventive Medicine, Regensburg University Medical Center, Regensburg, Germany; 7 Molecular Epidemiology Research Group, Max Delbrück Center for Molecular Medicine in the Helmholtz Association (MDC), Berlin, Germany; 8 Institute of Social Medicine, Epidemiology and Health Economics, Charité – Universitätsmedizin Berlin, corporate member of Freie Universität Berlin and Humboldt-Universität zu Berlin, Berlin, Germany; 9 GESIS – Leibniz-Institute for Social Sciences, Mannheim, Germany; 10 Leibniz Institute for Prevention Research and Epidemiology – BIPS, Bremen, Germany; 11 Institute for Medical Informatics, Biometry and Epidemiology, University Hospital Essen, Essen, Germany; 12 Federal Institute for Population Research, Wiesbaden, Germany; 13 Division of Cancer Epidemiology, DKFZ Heidelberg, Heidelberg, Germany; 14 Institute for Prevention and Cancer Epidemiology, Faculty of Medicine and Medical Center, University of Freiburg, Freiburg, Germany; 15 Department of Epidemiology, Fielding School of Public Health, University of California, Los Angeles, California USA

**Keywords:** Cohort Studies, Follow-Up studies, Prospective studies, Biostatistics, Data interpretation, statistical

## Abstract

The German National Cohort (NAKO) is an ongoing, prospective multicenter cohort study, which started recruitment in 2014 and includes more than 205,000 women and men aged 19–74 years. The study data will be available to the global research community for analyses. Although the ultimate decision about the analytic methods will be made by the respective investigator, in this paper we provide the basis for a harmonized approach to the statistical analyses in the NAKO. We discuss specific aspects of the study (e.g., data collection, weighting to account for the sampling design), but also give general recommendations which may apply to other large cohort studies as well.

## Introduction

The German National Cohort (NAKO, “NAKO Gesundheitsstudie”) investigates the causes, predictive factors, (pre-)clinical markers and functional health impairments underlying common chronic diseases, e.g., cardiovascular disease, cancer, diabetes, neurodegenerative/-psychiatric disorders, respiratory, and infectious diseases [1]. In 18 study centers across Germany, more than 205,000 women and men aged 19–74 years participated in a baseline examination between 2014 and 2019 [2]; a follow-up investigation is currently ongoing. The response proportion for the baseline examination was 17% [2]. The study center visits entail a face-to-face interview, completion of self-administered questionnaires, various physical examinations and assessments, as well as the collection of biospecimens, including blood, urine, feces, saliva and nasal swabs. A sub-sample of more than 57,000 participants followed an intensified examination program that included more in-depth physical and medical examinations. Between their study center visits, all participants are requested to answer questionnaires on their health status. Major self-reported diseases (cardiovascular disease, stroke, cancer, diabetes, depression, dementia) are then validated via physician contacts and pathology reports. Access to NAKO data for scientific use is open for all scientists according to the NAKO data use & access regulations.

This paper aims to provide the basis for a harmonized approach to the statistical analyses of NAKO data by pointing to specific aspects of the study (e.g., data collection, weighting to account for the sampling design), but also by giving some general recommendations which may also apply to other large cohort studies. Especially with reference to the large sample size, the late Sir D.R. Cox reminded us recently that “the size of the data does not remove the need for appropriate study design and statistical analysis.” [3].

This paper was jointly drafted by members of the expert group “Statistical Analysis” of NAKO, a group of statisticians and epidemiologists with considerable experience in methods and analysis of epidemiologic data. The authors respect the freedom of science and emphasize that each researcher is responsible for his/her own statistical analysis. As such, this paper only provides recommendations and refrains from prescriptive mandates. Most recommendations and comments can also be found in numerous tutorial papers, especially from the STRATOS initiative ([4], stratos-initiative.org), which provides accessible and accurate guidance in the analysis of observational studies, the “Education Corner” of the International Journal of Epidemiology [5], or the “Practice of Epidemiology” series in the American Journal of Epidemiology [6]. For the reporting of analyses, we refer to the STROBE statement ([7], strobe-statement.org).

## Data management and data quality

NAKO data were collected, whenever possible, in a standardized Electronic Case Report Form (ECRF) web application with data entry forms generated from the central data dictionary. Entered values were immediately stored in the centralized database to avoid loss of data and to conduct immediate data validation (e.g., plausibility checks). ECRFs could not be completed without filling all fields or submitting a reason for aborting an examination. Output of diagnostic devices was uploaded to the centralized database, parsed and validated by an integrated data transfer application. In cases of technical failures and as preliminary workaround, paper forms were used to collect data for later entry into the ECRF application.

Data quality assessments and data cleaning for the different NAKO examinations were performed by scientists individually responsible for the respective modules, competence units (for more complex biomedical data), or by expert groups of the NAKO. All persons involved in plausibility checks and data cleaning were requested to check the data for completeness and expected distributions of variables. In case of deviations, recommendations for the handling of implausible values were given; if necessary, the original variables were replaced by the corrected ones. If implausible values were considered possible but not convincing these values were not corrected but highlighted in module-specific quality reports. In addition, the respective experts in charge decided on and defined derived variables, which have been added to the datafiles. Missing values were coded in accordance with the reason for missingness, if known.

All information on variables (“metadata”), including important results from data quality assessments, is collected in the NAKO data dictionary which is publicly accessible through the NAKO Transfer Hub (transfer.nako.de, registration required).

Reliability of measurements and correcting for measurement error is an important aspects in all large cohort studies and also for NAKO. Thus, a calibration study was conducted where n = 5,903 participants from the baseline examination were re-investigated within 1 to 12 months. This calibration study is currently being analysed by a combination of regression calibration approaches and longitudinal data analysis methods, and will give recommendations on calibrating measurements in regression analyses.

## Weighting factors for survey design and/or non-response

Epidemiologic data that are based on a random sample allow valid statistical inference regarding the underlying target population. Since not all population groups are equally accessible and not all persons invited to a study take part, the composition of the final study sample will in general deviate from the target population, potentially leading to biased inference. Weighting of data changes the relative impact of an observed sample element to generate estimates that are closer to the true value of the target population than the unweighted estimates [8, 9]. To this end, a weight ${w}_{i}$ for a sample element $i$ is constructed that can be used for every analysis to provide unbiased or at least less biased estimates. Following Gabler et al., the main reasons to weigh a data set are (a) to reduce potential biases due to unequal inclusion probabilities of sampling elements, (b) to reduce potential biases due to nonresponse and (c) to increase the precision of an estimate by retroactive stratification [10]. For these purposes, design weighting (a) and calibration (b, c) are generally distinguished.

Design weights are recommended whenever the design of a study sample causes unequal inclusion probabilities for sample elements and researchers are interested in estimates of particular subpopulations. They are generally calculated by the inverse of an element’s inclusion probability. We recommend employing design weights for inferential estimations.

Calibration weights, on the other hand, use auxiliary information available for the target population (e.g., socioeconomic data from official population statistics) to adjust the study sample in order to align the sample’s (marginal) distribution to that of the population when using the calibrated estimator. The actual benefit of calibration weights (i.e., the reduction in bias), and therefore researchers’ decisions whether or not to use them in a particular analysis, critically depends on the variables available for calibration, the underlying nonresponse mechanism, and of course the particular variable of interest [8, 9, 11].

The use of correction weights is generally not advised when estimating complex models, because models usually come with assumptions that might be hard to satisfy [12], but exceptions to this general rule are known, see, e.g., Hernán/Robins [13] for causal modeling.

In NAKO, population statistics from the German Federal Statistical Office are used to calculate design and calibration weights. Correction weights, as well as information on their use and its reporting are provided with the NAKO data set, since it is known that the use of weights can vary considerably across publications even for the same data set [14]. Since NAKO is performed in 18 study centers that were not randomly selected and the respective local regulations led to slightly different ways to arrive at the final population sample, weights have been calculated for each study center separately. Thus, the underlying weighting strategy does not target Germany as a whole, but the target populations of individual study regions.

## Mortality follow-up

Vital status (VS) and causes of death (CoD) as documented on the death certificate (DC) cannot be retrieved from a central registry in Germany. Therefore, the mortality follow-up in the NAKO is case-by-case tracked by the “Competence Center Mortality Follow-Up” (MoFU).

Standard for the CoD documentation is the WHO “International Form of Medical Certificate of Cause of Death” [15]. In view of the four goals of any mortality follow-up - authenticity, compatibility, completeness and generalizability - three versions of the CoD diagnoses are provided by the MoFU, offering full choice options to users. The first gives CoD diagnoses in ICD codes exactly as on the DC. In the second version those ICD codes are potentially rearranged by the coding software IRIS [16], which is used in all European (and many more) National Statistical Offices, thereby making the first CoD diagnoses version comparable to official mortality statistics. For a third version the MoFU retrieves additional CoD information from attending physicians, hospices, law-enforcement agencies, next-of-kin, etc. In 30% of deaths there are noteworthy, in 10% substantial differences between CoD information on the DC (even after IRIS rearrangement) and third version CoD information. In addition, the third version allows a longer look back in the case history – certifying physicians often neglect according ICD codes.

For follow-up on morbidity of study participants, a combination of methods is used, including active and passive follow-up procedures. Active follow-up includes written health follow-up questionnaires sent to participants every 2–3 years with subsequent contacts with the participants’ treating physicians and hospitals and ascertainment of events by medical records. Passive follow-up procedures include use of secondary data, e.g., from cancer registries or health insurance companies.

## Missing values/Multiple imputation

Different strategies are available to deal with missing values, and the choice depends on three factors: (a) the degree of missingness, (b) the nature of missingness, and (c) the intended use of the variable in question.

- Degree of Missingness

In the NAKO, the degree of item missingness ranges from 0 to 25% per variable. For such variables an available case method is generally the appropriate analytic strategy. Generally, there are less missing values (often 0 to 1%) in variables obtained in the face-to-face interview, some more missing values (usually 5 to 15%) on the self-administered touch screen questionnaires and a wide range of missing values in the variables obtained from physical and medical examinations. There are special cases of missingness by design, e.g., for variables only assessed in participants with the intensified examination program, where the degree of missingness could be 75% or more. For such variables, when analysis is not restricted to the respective sub-cohort, an available case method is generally the appropriate analytic strategy.

(b) Nature of Missingness

When deciding on how to treat missing values, it is imperative to evaluate whether the values are missing completely at random (MCAR), missing at random (MAR), or not missing at random (NMAR) [17, 18]. This is often not obvious and assumptions have to be made. Of note, an assumption of missingness completely at random can almost never be made. Few missing values are truly random [17, 18], but for values missing by design the assumption of randomness is likely reasonable if measurements are taken in a random subgroup of individuals. Investigators might want to explore missing distributions by age, sex, study center, and other covariates. The NAKO has generated a detailed list of missing categories providing guidance to investigators with information about the nature of missingness. These categories are: do not know; blank, implausible value; value could not be derived, variable/module was not assessed (reason provided, e.g. participant refusal, contraindicated, skipped due to lack of time, instrument malfunction), missing by design. The coding of the missing variables is available from the NAKO code book.

(c) Intended Use of Variable

The strategy of how to approach missing values depends on whether the variable is used for prevalence or incidence estimations, whether it represents an exposure or outcome of interest in a regression model, or whether it is a confounder or covariate in a regression model [17–19]. For estimating prevalence and incidence, multiple imputation is probably the best approach. If model building is the objective, both primary exposure and outcome variables should follow an available case method and not be imputed [18, 19]. Potential confounders and other covariates that are not clearly NMAR can be imputed using a single imputation method which is simple to perform and will provide appropriate variance estimates given the sample size and the fraction of missing values of NAKO. Only in case of an obvious NMAR situation, an available case method should be preferred for covariates [18]. Table 1 displays an exemplary decision on how to handle missing values in NAKO. Of note, this is not a blanket recommendation but more an example to guide decision making, and choices will vary depending on the research question. It is important that the handling of missing values is reported in an appropriate manner in the methods section of the manuscript. Furthermore, to follow the guidelines below, the proportion of missing values should not be severe. The severity of missingness is a multidimensional problem that depends on a multitude of characteristics, such as variable’s distribution, the research question, the type of nonresponse, the data’s covariance structure, etc., and in specific situations even a small number of missing values may result in a distorted estimate.

**Table 1:** Treatment of missing values by nature of missingness and research question as appropriate in NAKO

Variable and Analysis/ Missing Type	Missing Completely at Random (MCAR)	Missing at Random (MAR)	Not Missing at Random (NMAR)
Incidence/Prevalence	Available Case Analysis	(Multiple-) Imputation	Complete Case Analysis (reporting restrictions)
Outcome or Exposure in a regression model	Available Case Analysis	Available Case Analysis	Complete Case Analysis (reporting restrictions)
Covariate in a regression model	Available Case Analysis	(Single-) Imputation	Available Case Analysis (reporting restrictions)

## Covariate/Confounder selection

In principle, two statistical modelling approaches in epidemiology with different philosophies can be distinguished: causal modelling and individual prediction modelling. In NAKO, in agreement with many recent cohort studies, the focus is on causal modelling approaches. Causal inference is based on potential outcomes for which four fundamental identification conditions are required including exchangeability, positivity, counterfactual consistency, and no interference [20, 21]. Causal diagrams also known as directed acyclic graphs (DAGs) can answer interventional and counterfactual questions. A confounder is defined as any variable that can close a backdoor path between an exposure and an outcome [22]. This modern definition is about to replace the well-known previous definition that a confounder simply represents a variable that is associated with the outcome and with the exposure of interest. DAGs, and especially the directions of the paths between covariates and exposure or outcomes or between covariates are drawn based on prior knowledge. Based on a DAG, a minimal sufficient adjustment set of confounders is selected and used as covariates in regression analyses. It is important to note that the wealth of variables in large cohorts may also lead to over-fitting and adjustment for mediating variables in cases of not correctly specified DAGs. Of course, the latter might results from errors of respective researchers, but also are likely to occur based on limited knowledge at the time of DAG generation.

If there is little prior knowledge about relations between variables, VanderWeele [23] suggests to adjust for each covariate that is either a cause of the exposure or the outcome or is a cause of both the exposure and the outcome. In contrast to smaller studies, NAKO enables extensive adjustment or even stratification for many confounders simultaneously with an acceptable loss of statistical precision. In any case, in a multicenter study like NAKO it is of central interest how to deal with the center effect in regression analyses. In principle, we consider the center effect as a regular covariate, and the decision about adjusting or not adjusting for it depends on its anticipated role as a confounder or a non-confounder.

The covariate selection should be made explicit in an a priori analysis plan independently of the method used and should not be based on the statistical significance of p-values in (bivariate) group comparisons. Likewise, an increase or decrease in effect size of the exposure on the outcome due to further adjustment for a covariate in a regression model is not necessarily an indication that the covariate is a confounder, because colliders (common effects of exposure and outcome) or mediators (effect of exposure, cause for the outcome) can also produce a change in the effect size. Furthermore, the non-collapsibility property of the odds ratio can result in a change in effect estimate that is unrelated to confounding [24].

## Dichotomization/Categorization

In epidemiologic studies, many variables are obtained on a quantitative (or continuous) as opposed to a categorical scale, either by direct measurement (e.g., age) or by combining information from several variables to a new composite variable (e.g., BMI). When analyzing data from NAKO the following should be considered: In descriptive analyses, continuous variables should be presented providing the mean or median, a measure of spread (standard deviation, interquartile range, or range), or with a figure, e.g., a histogram. The appropriate handling of a continuous covariate in a regression model is less clear. Several methods are available with specific advantages and disadvantages (Table 2).

**Table 2:** Methods to model continuous covariates in regression models

Method	Advantages	Disadvantages
Categorization into two or more groups	Easy to communicate; Enables comparison to earlier studies which use the same cutpoints; Can reflect disease stages and potential therapeutic consequences	Information loss; Induces biologically unplausible step functions as dose-response relationships; Inflation of α- and β-errors; Potentially arbitrary selection of cutpoints
Leaving the variable as measured	No information loss	(In general) Assumption of linear relation
Transformation of the variable according to a specified procedure (e.g., fractional polynomials)	Non-linear and non-monotone relations possible; Statistical tools available; High statistical power	Arbitrary or data-driven choice of transformation
Spline regression	Allows complex dose-response relationships; “Letting the data speak for themselves”	Data driven method; Danger of overfitting; Difficult in comparison across studies

When a continuous variable is used to adjust for confounding (e.g., “adjusted for age”) in the common way, only the linear component of the confounder is accounted for giving rise to potential residual confounding. Conversely, when the variable is categorized or dichotomized, not only a (sometimes tremendous) loss of information is inducted, but model assumptions like step-wise constant effects on the outcome are implicitly made; this might again introduce residual confounding, which may lead to both a loss of power and inaccurate estimation [25, 26]. Therefore, we do not recommend the categorization of continuous covariates in the modelling stage.

On the other hand, the dichotomization of a quantitative variable in an association model can be warranted, when this reflects a pre-defined disease status (or disease stage in the case of multiple categories) defined by clinical guidelines. This is relevant when the covariate-disease status may be linked to therapeutic consequences: for example, when hypertension is associated with an x-fold increased risk of cardiovascular disease, then individuals with hypertension are subject to potential therapy and the risk increase is the impact on disease that may be avoided. A categorization can also be warranted to compare NAKO results with those from previous studies. In any case, the use of categories for quantitative variables as covariates should be justified by sound scientific or clinical/public health arguments [27] and accompanied by statistically more appropriate methods.

Ideally, the functional form of the confounder’s effect on the outcome is to be evaluated in the modelling procedure and is best accounted for as fully as possible, e.g., by fractional polynomials or splines [28, 29]. For variables with a semi-continuous distribution (spike at zero), for example, dose of lifetime smoking when never-smokers are included, methods are available using an expanded fractional polynomial procedure [30].

## Estimation and quantification of associations

In risk factor epidemiology, the key objective is to estimate the association between an exposure variable and a disease outcome along with the quantification of the strength and direction of such an association. For quantification, we consider a p-value insufficient to describe empirical evidence of an association because it confounds the size of the effect with its precision. In NAKO, due to the large sample size of the study, nearly every p-value will be dramatically low and might be labelled “statistically significant”. We thus recommend to use p-values only in specific situations and always, at least if possible, with the respective parameter estimate and a measure of precision. Preferred is a 95% confidence interval even though it might be argued that in large cohort studies like NAKO 95% confidence intervals might be too narrow to be of value or may suggest a level of precision not supported by the data or the measurement process itself. Situations where a p-value indeed might be reported are those where it is difficult to provide parameter estimates, e.g., for a test for trend or when testing the fit of a smooth/flexible regression fit against a linear or a null fit. However, these p-values should be accompanied with meaningful graphs to describe the situation under study. In any case it is important to communicate and interpret p-value correctly, that is, not as a probability of the null hypothesis being true [31], but the probability that the observed (or a more extreme) result would have occurred if the null hypothesis had been true.

There is considerable debate about the usage of p-values in a dichotomized fashion, i.e., in the judgement of an association being “statistically significant” and “not statistically significant” at arbitrary cutpoints [32]. Statistical significance is frequently erroneously equated with epidemiologic or clinical relevance, and a statistically significant result is considered epidemiologically important, whereas a statistically non-significant result is considered as not being important.

In summary, we recommend sparse utilization and cautious interpretation of p-values in data sets like NAKO. Dichotomization in statistically significant and non-significant results should be entirely abandoned. Finally, we also encourage the reporting of null associations, which are frequently omitted from the scientific literature but are equally important as non-null associations.

## Subgroup analyses

Subgroup or stratified analyses are an important tool in epidemiology, for instance, to account for pathophysiological heterogeneity or differences in risk profiles [33]. However, they come with certain pitfalls if not applied with care. At worst, unplanned post-hoc analysis without clear justification can lead to misleading and even wrong conclusions [34]. The NAKO with its wealth of variables will lend itself to pursue subgroup analyses. Note that, although we use a dichotomized interpretation of p-values in the following remarks since it is still common in these types of analyses, the aforementioned caution against it nevertheless applies.

Defining subgroups of participants based on certain characteristics and investigating differential exposure effects or occurrences of outcomes can lead to important findings, but, with a multitude of possible comparisons, possibly also to false positive results due to inflated overall α-levels. Therefore, instead of testing large numbers of group differences post-hoc, the number of comparisons should be limited, and they should be defined and justified a priori. Results from post-hoc tests should always be reported together with the number of tests carried out and the information whether and which adjustments were made for multiple comparisons [35].

Moreover, comparisons between subgroups should not be solely based (and interpreted) on the fact that separate tests reached significance in one group but not in the other, because the difference between a significant and a non-significant test result is not necessarily significant itself [36]. Instead, group differences should only be reported if relevant interaction between group and exposure effects is present in addition to significant main effects [33].

When exploring subgroup analyses in NAKO these should be defined a priori based on pathophysiological principles, carefully justified, adequately sized, and limited to few endpoints and subgroups of interest. If group differences are observed post-hoc even after accounting for multiplicity, they should be interpreted carefully regardless of their statistical significance and all comparisons made and endpoints analyzed should be reported.

More recent approaches to subgroup analyses include Bayesian methods, for instance, for the evaluation of heterogeneity of treatment effects [37] or subgroup analysis with hierarchical models [38], as well as machine learning approaches [39, 40].

## Conclusion and outlook

In summary, we have emphasized some specific characteristics in the NAKO data collection and sample composition that call for appropriate analytic methods, such as the use of weights. Moreover, we have highlighted other considerations for data analysis, which we hope will be helpful for individual researchers and will provide the basis for a unified approach to statistical analyses in NAKO and other large cohort data.

With respect to the future, an important aspect for the next funding period is genotyping of all 205,000 NAKO participants using a genome-wide single nucleotide polymorphism array. This will allow, besides the standard application of genomic information, also more advanced methodological approaches, like polygenic risk scores or Mendelian randomization analyses. The ‘large data’ will allow to connect multiple layers of biological information with new computational approaches (e.g., artificial intelligence) and will contribute to an enhanced understanding of human physiology and pathomechanisms.
